# Supplementary material for: Benchmarking protein language models for protein crystallization
Source: Sci Rep. 2025 Jan 18;15:2381. doi: 10.1038/s41598-025-86519-5 (PMC11743144; doi:10.1038/s41598-025-86519-5)
Supplement: Supplementary file 1 — Supplementary Information. [file 41598_2025_86519_MOESM1_ESM.pdf]

Benchmarking Protein Language Models for Protein Crystallization using TRILL

Raghvendra Mall[1,*], Rahul Kaushik[1], Zachary A. Martinez[2], Matt W. Thomson[2], and Filippo Castiglione[1,3,*]

[1]Biotechnology Research Center, Technology Innovation Institute, P.O. Box 9639, Abu Dhabi, United Arab Emirates
[2]Division of Biology and Bioengineering, California Institute of Technology, Pasadena, 91125, California, United States of America
[3]Institute for Applied Computing, National Research Council of Italy, Rome, 00185, Italy
[*]Corresponding authors: raghvendra.mall@tii.ae, filippo.castiglione@tii.ae

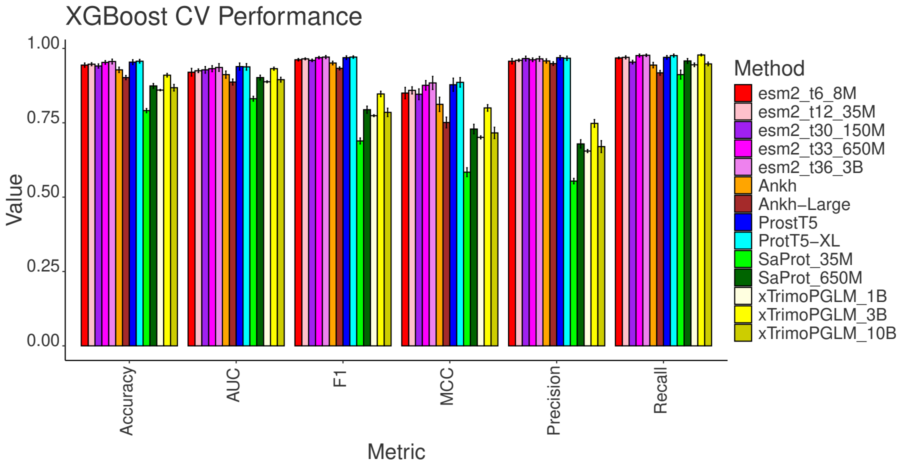

Fig. S1: Cross-validation performance of XGBoost classifiers built from different PLMs.

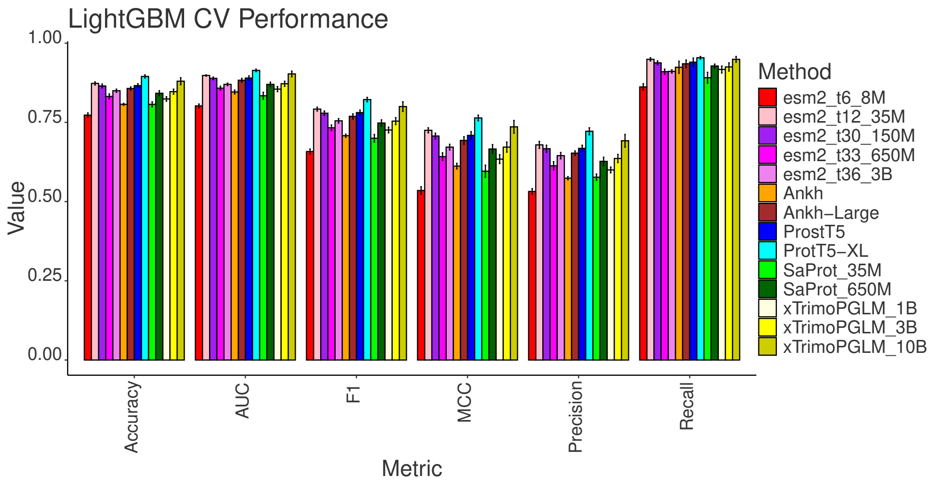

Fig. S2: Cross-validation performance of LightGBM classifiers built from different PLMs.

Model	Method	F1	ACC	MCC	Prec	Rec	AUPR	AUC
Balanced Test Set								
ESM2 T6-8M	MLPClassifier	0.707	0.72	0.482	0.875	0.511	0.86	0.719
ESM2 T12-35M	MLPClassifier	0.7	0.715	0.48	0.888	0.49	0.855	0.715
ESM2 T30-150M	MLPClassifier	0.791	0.795	0.614	0.907	0.657	0.92	0.795
ESM2 T33-650M	MLPClassifier	0.773	0.778	0.586	0.906	0.62	0.904	0.778
ESM2 T36-3B	MLPClassifier	0.76	0.767	0.568	0.906	0.595	0.901	0.767
Ankh-Large	MLPClassifier	0.751	0.757	0.543	0.879	0.595	0.877	0.757
Ankh	MLPClassifier	0.733	0.74	0.507	0.854	0.578	0.868	0.74
ProstT5	MLPClassifier	0.776	0.78	0.575	0.865	0.661	0.894	0.779
ProtT5-XL	MLPClassifier	0.796	0.8	0.62	0.902	0.671	0.904	0.799
SaProt-35M	MLPClassifier	0.746	0.752	0.53	0.866	0.595	0.878	0.752
SaProt-650M	MLPClassifier	0.75	0.755	0.533	0.858	0.611	0.878	0.755
xTrimoPGLM-1B	MLPClassifier	0.794	0.796	0.604	0.871	0.692	0.897	0.795
xTrimoPGLM-3B	MLPClassifier	0.752	0.758	0.545	0.881	0.596	0.876	0.758
xTrimoPGLM-10B	MLPClassifier	0.783	0.787	0.593	0.885	0.658	0.891	0.786
SP_final Test Set								
ESM2 T12-35M	MLPClassifier	0.59	0.624	0.401	0.928	0.432	0.756	0.688
ESM2 T30-150M	MLPClassifier	0.781	0.764	0.57	0.926	0.676	0.828	0.793
ESM2 T33-650M	MLPClassifier	0.795	0.781	0.61	0.953	0.682	0.849	0.813
ESM2 T36-3B	MLPClassifier	0.776	0.764	0.586	0.951	0.655	0.838	0.8
ESM2 T6-8M	MLPClassifier	0.581	0.616	0.382	0.913	0.426	0.747	0.679
Ankh-Large	MLPClassifier	0.749	0.734	0.522	0.913	0.635	0.807	0.767
Ankh	MLPClassifier	0.669	0.667	0.415	0.879	0.541	0.762	0.708
ProstT5	MLPClassifier	0.764	0.747	0.54	0.915	0.655	0.815	0.777
ProtT5-XL	MLPClassifier	0.783	0.764	0.564	0.918	0.682	0.825	0.791
SaProt-35M	MLPClassifier	0.678	0.671	0.415	0.872	0.554	0.762	0.71
SaProt-650M	MLPClassifier	0.73	0.722	0.516	0.927	0.601	0.806	0.761
xTrimoPGLM-1B	MLPClassifier	0.789	0.768	0.566	0.912	0.696	0.824	0.792
xTrimoPGLM-3B	MLPClassifier	0.717	0.713	0.51	0.935	0.581	0.805	0.757
xTrimoPGLM-10B	MLPClassifier	0.781	0.764	0.57	0.926	0.676	0.828	0.793
TR_final Test Set								
ESM2 T6-8M	MLPClassifier	0.646	0.788	0.534	0.841	0.524	0.617	0.733
ESM2 T12-35M	MLPClassifier	0.628	0.785	0.531	0.868	0.492	0.615	0.724
ESM2 T30-150M	MLPClassifier	0.78	0.855	0.685	0.885	0.698	0.729	0.822
ESM2 T33-650M	MLPClassifier	0.755	0.844	0.663	0.9	0.65	0.714	0.804
ESM2 T36-3B	MLPClassifier	0.748	0.839	0.651	0.886	0.647	0.704	0.799
Ankh-Large	MLPClassifier	0.719	0.818	0.602	0.839	0.628	0.665	0.779
Ankh	MLPClassifier	0.678	0.796	0.552	0.816	0.58	0.628	0.752
ProstT5	MLPClassifier	0.73	0.818	0.601	0.808	0.666	0.662	0.787
ProtT5-XL	MLPClassifier	0.772	0.848	0.668	0.867	0.695	0.715	0.816
SaProt-35M	MLPClassifier	0.683	0.799	0.559	0.82	0.586	0.633	0.755
SaProt-650M	MLPClassifier	0.682	0.796	0.552	0.807	0.591	0.628	0.754
xTrimoPGLM-1B	MLPClassifier	0.756	0.836	0.641	0.84	0.687	0.693	0.805
xTrimoPGLM-3B	MLPClassifier	0.714	0.817	0.6	0.846	0.618	0.664	0.776
xTrimoPGLM-10B	MLPClassifier	0.749	0.835	0.64	0.856	0.666	0.693	0.8

Table S1: Performance of MLP classifiers on the balanced, SP_final and TR_final test sets.

**Methods**	**Prot-142**	**Prot-630**	**Prot-851**	**Prot-1120**	**Prot-1302**
DeepCrystal	0.805	0.642	0.999	**0.448**	0.878
CLPred	0.771	**0.485**	0.919	0.71	0.557
ESM2 T6-8M	0.718	0.823	0.93	0.551	0.825
ESM2 T12-35M	0.718	0.823	0.93	0.551	0.825
ESM2 T30-150M	0.903	0.633	0.935	0.902	0.527
ESM2 T33-650M	0.576	0.525	0.582	0.576	0.505
ESM2 T36-3B	0.846	0.622	0.994	0.971	0.646
Ankh	0.868	0.592	0.981	0.568	0.737
Ankh-Large	0.689	0.507	0.975	0.577	0.723
ProstT5	0.722	0.635	0.991	0.619	0.565
ProtT5-XL	0.946	0.722	0.972	0.955	0.548
Consensus	0.776	0.654	0.921	0.697	0.656

Table S2: Comparison of prediction probability of PLM based classifiers with DeepCrystal and CLPred. For the 5 candidate proteins identified by our Consensus framework as crystallizable, 4 out of the 5 were identified by both DeepCrystal and CLPred as crystallizable.

**Models**	**pLDDT**	**TM Score**	**GDT-TS**	**GQ Score**
Prot-142 AF2-Model 1	61.14	0.71	0.66	0.39
Prot-142 AF2-Model 2	57.74	0.68	0.63	0.37
Prot-142 AF2-Model 3	56.11	0.79	0.64	0.38
Prot-142 AF2-Model 4	55.27	0.66	0.61	0.38
Prot-142 AF2-Model 5	55.34	0.62	0.61	0.37
Prot-142 Final Model	NA	0.83	0.75	0.44
Prot-630 AF2-Model 1	65.72	0.74	0.68	0.40
Prot-630 AF2-Model 2	62.87	0.72	0.66	0.39
Prot-630 AF2-Model 3	62.17	0.71	0.66	0.39
Prot-630 AF2-Model 4	62.12	0.71	0.65	0.38
Prot-630 AF2-Model 5	60.18	0.68	0.64	0.38
Prot-630 Final Model	NA	0.83	0.84	0.41
Prot-851 AF2-Model 1	73.84	0.65	0.59	0.44
Prot-851 AF2-Model 2	72.11	0.64	0.59	0.44
Prot-851 AF2-Model 3	69.63	0.61	0.58	0.43
Prot-851 AF2-Model 4	64.72	0.57	0.56	0.42
Prot-851 AF2-Model 5	64.61	0.58	0.56	0.40
Prot-851 Final Model	NA	0.65	0.63	0.45
Prot-1120 AF2-Model 1	61.91	0.66	0.62	0.41
Prot-1120 AF2-Model 2	59.36	0.67	0.59	0.39
Prot-1120 AF2-Model 3	58.75	0.65	0.60	0.40
Prot-1120 AF2-Model 4	58.37	0.66	0.61	0.39
Prot-1120 AF2-Model 5	55.29	0.64	0.58	0.37
Prot-1120 Final Model	NA	0.80	0.72	0.44
Prot-1302 AF2-Model 1	60.36	0.75	0.78	0.32
Prot-1302 AF2-Model 2	59.92	0.74	0.77	0.31
Prot-1302 AF2-Model 3	59.86	0.75	0.78	0.31
Prot-1302 AF2-Model 4	59.81	0.74	0.75	0.30
Prot-1302 AF2-Model 5	56.45	0.72	0.74	0.28
Prot-1302 Final Model	NA	0.82	0.88	0.35

Table S3: A summary of the quality assessment of the predicted model structure for the designed proteins. The pLDDT scores were directly adopted from AlphaFold2 predictions, while TM Score and GDT-TS were predicted through ProFitFun, and GQ (Global Quality) Score was calculated by ModFold.

Protein	Cellular Component	Biological Process	Molecular Functions
Prot-142	GO:0005737, GO:0016020, GO:0110165	GO:0006810, GO:0008152, GO:0009058, GO:0044237, GO:0044238, GO:0044249, GO:0044281, GO:0050794, GO:1901564	
Prot-630	GO:0005737, GO:0110165	GO:0008152, GO:0009987, GO:0044237, GO:0044238	GO:0003824
Prot-851	GO:0005886, GO:0016020, GO:0071944, GO:0110165	GO:0006810, GO:0009987, GO:0044237	GO:0008324, GO:0015291, GO:0015318, GO:0022804, GO:0022890
Prot-1120	GO:0005737, GO:0016020, GO:0110165	GO:0006793, GO:0006796, GO:0008152, GO:0044237, GO:0044238, GO:0050794, GO:1901564	GO:0003824, GO:0005544, GO:0016740, GO:0016772, GO:0032559, GO:0035639, GO:0036094, GO:0042578, GO:0043167, GO:0043168, GO:0097159, GO:0097367, GO:1901265, GO:1901363
Prot-1302	GO:0005737, GO:0016020, GO:0071944, GO:0110165	GO:0006793, GO:0006810, GO:0006811, GO:0008152, GO:0009058, GO:0009141, GO:0009142, GO:0044237, GO:0044238, GO:0044249, GO:0044281, GO:0046390, GO:0072521, GO:0072522, GO:0098655, GO:0098660, GO:0098662, GO:1901135, GO:1901137	GO:0005216, GO:0005524, GO:0015318, GO:0016740, GO:0016772, GO:0016776, GO:0022803, GO:0022890, GO:0035639, GO:0036094, GO:0043167, GO:0043168, GO:0097159, GO:0097367, GO:1901265, GO:1901363

Table S4: Sequence and structure based functional annotation of selected designed protein.

Model	Method	Train Loss	Train Acc	Train F1	Valid Loss	Valid Acc	Valid F1
ESM2 T30-150M	CNN + AVG Embed	0.22 ± 0.07	0.89 ± 0.04	0.77 ± 0.09	0.33 ± 0.03	0.82 ± 0.02	0.67 ± 0.01
ESM2 T36-3B	CNN + AVG Embed	0.24 ± 0.01	0.88 ± 0.0	0.75 ± 0.01	0.32 ± 0.0	0.82 ± 0.0	0.65 ± 0.01
ProstT5	CNN + AVG Embed	0.24 ± 0.02	0.87 ± 0.01	0.73 ± 0.03	0.34 ± 0.01	0.82 ± 0.0	0.68 ± 0.0
ESM2 T30-150M	LSTM + AVG Embed	0.24 ± 0.06	0.87 ± 0.04	0.7 ± 0.11	0.33 ± 0.04	0.83 ± 0.01	0.65 ± 0.0
ESM2 T36-3B	LSTM + AVG Embed	0.25 ± 0.0	0.87 ± 0.0	0.72 ± 0.01	0.32 ± 0.0	0.83 ± 0.0	0.64 ± 0.0
ProstT5	LSTM + AVG Embed	0.25 ± 0.05	0.87 ± 0.03	0.7 ± 0.08	0.34 ± 0.01	0.83 ± 0.01	0.65 ± 0.0

Table S5: Benchmarking performance of residue-level CNN and LSTM models built on top of embeddings obtained from the optimal PLMs for protein crystallization propensity prediction task.

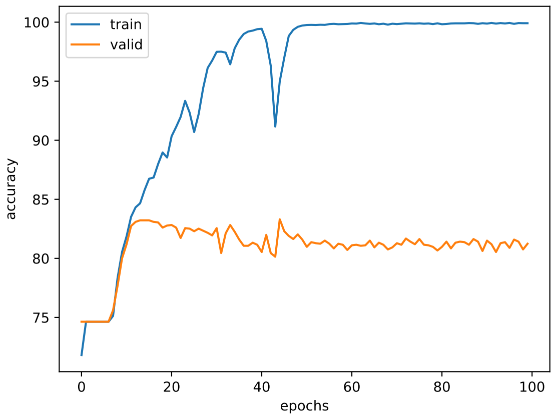

(a) ESM2 T30-150M best CNN model accuracy.

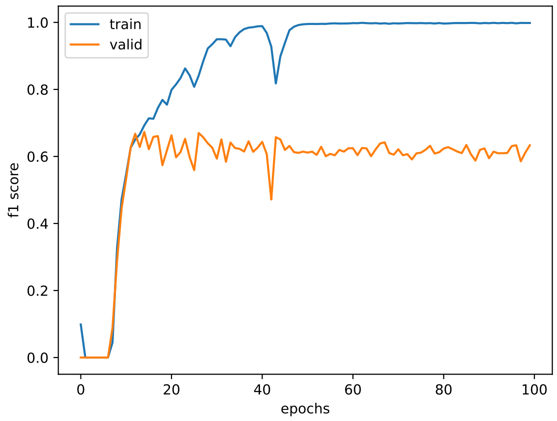

(b) ESM2 T30-150M best CNN model F1 score.

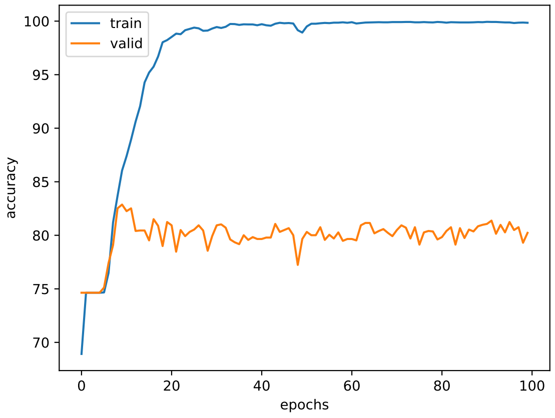

(c) ESM2 T36-3B best CNN model accuracy.

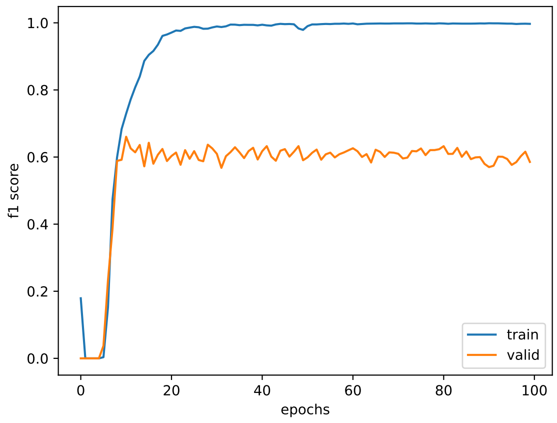

(d) ESM2 T36-3B best CNN model F1 score.

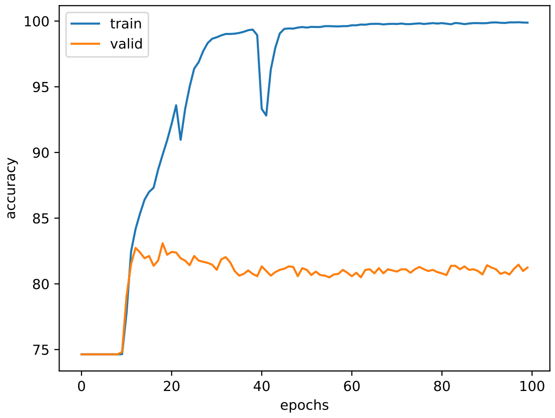

(e) ProstT5 best CNN model accuracy.

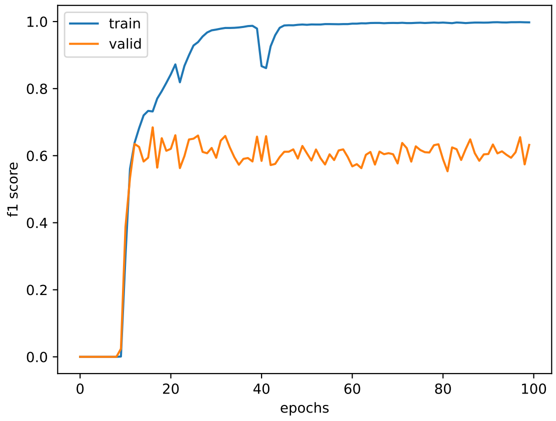

(f) ProstT5 best CNN model F1 score.

Fig. S3: Performance comparison of ESM2 T30-150M, ESM2 T36-3B and ProstT5 for optimal CNN hyper-parameter for crystallization propensity task.

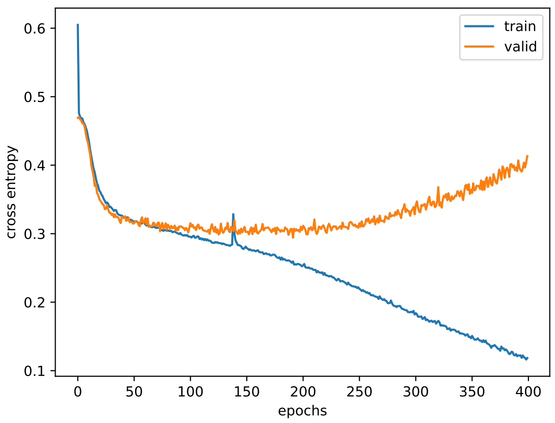

(a) ESM2 T30-150M best LSTM model loss.

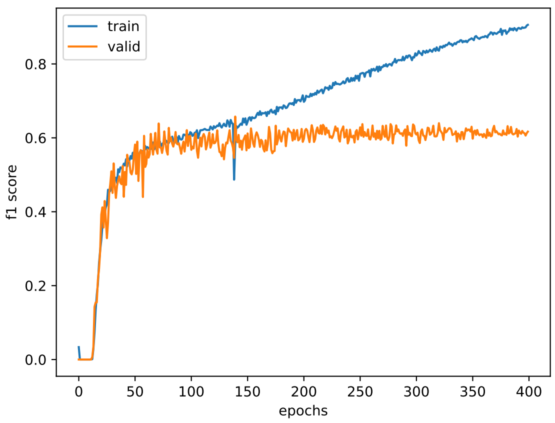

(b) ESM2 T30-150M best LSTM model F1 score.

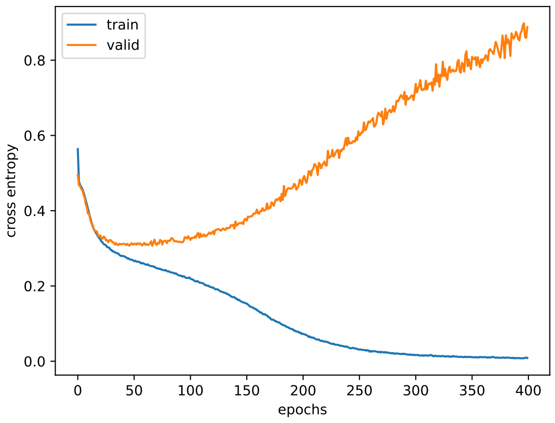

(c) ESM2 T36-3B best LSTM model loss.

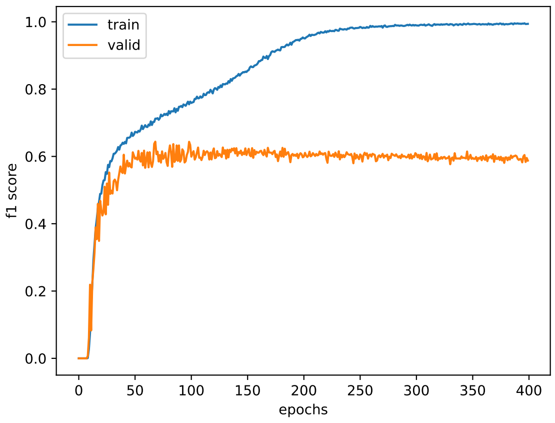

(d) ESM2 T36-3B best LSTM model F1 score.

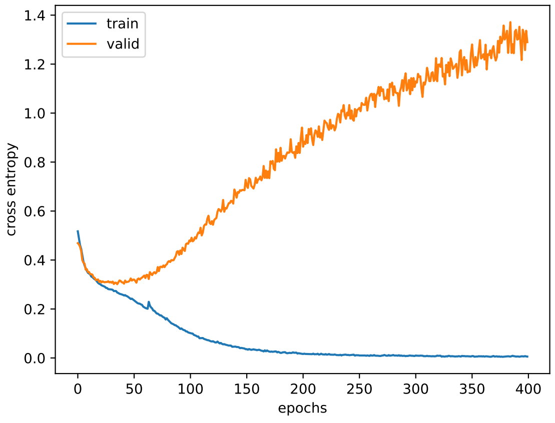

(e) ProstT5 best LSTM model loss.

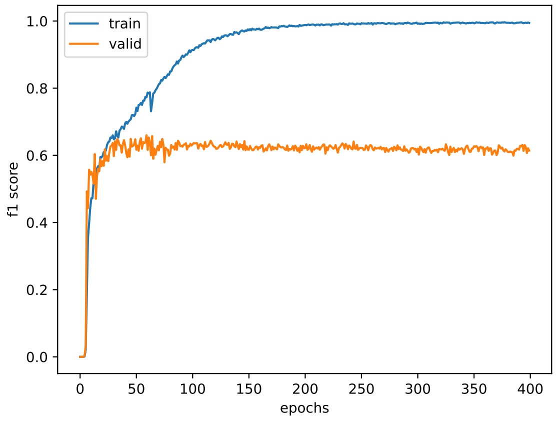

(f) ProstT5 best LSTM model F1 score.

Fig. S4: Performance comparison of ESM2 T30-150M, ESM2 T36-3B and ProstT5 for optimal LSTM hyper-parameter for crystallization propensity task.
